# Innovative air-impingement jet drying of red cabbage: Kinetic description and prediction of the degradation of cyanidin-3-diglucoside-5-glucoside and cyanidin

**DOI:** 10.1016/j.fochx.2022.100422

**Published:** 2022-08-10

**Authors:** Wenfeng Li, Guangfeng Gou, Yanling He, Si Tan

**Affiliations:** School of Life Science and Biotechnology, Yangtze Normal University, Chongqing 408100, China

**Keywords:** Anthocyanin, Air-impingement jet drying, *Brassica oleracea* L. var. *capitata*, Degradation, Kinetics

## Abstract

Red cabbage (RC) is a vegetable rich in anthocyanins. This study analyzed comparative applications of RC hot air drying (HAD) at 60 °C, which took 810 min, and RC air-impingement jet drying (AIJD) at 60 °C, which took 630 min. The results indicated that AIJD retained a better color in the RC than HAD. Ultra-high performance liquid chromatography coupled with triple quadrupole mass spectrometry (UHPLC-QqQ-MS/MS) analysis of the RC showed that 28 anthocyanins and one anthocyanidin (cyanidin (C)) were derived from cyanidin-3-diglucoside-5-glucoside (C3dG5G). Multivariate analysis revealed that AIJD degraded the C3dG5G and C in the RC, which were revealed as 1.5-order and 2-order reactions, respectively. Moreover, the AIJD-induced degradation of C3dG5G and C were non-spontaneous and endothermic reactions. These kinetic equations could also be applied to predict the content of C3dG5G and C during degradation. Therefore, the results of this study could be beneficial in improving the AIJD of RC for more effective preservation.

## Highlights

• AIJD was better than HAD in the drying of RC.
• Twenty-eight anthocyanins and one anthocyanidin were found in the RC.
• C3dG5G and C degradations were non-spontaneous and endothermic reactions.
• C3dG5G and C degradations followed 1.5- and 2-order kinetic equations, respectively.

## Introduction

Dehydrated cabbage is an important raw material for vegetable bags of instant noodles and similar foods in China. Red cabbage (*Brassica oleracea* L. var. *capitata*) (RC) may be a better choice than common cabbage since it is also an important source of anthocyanins (Sakulnarmrat, Wongsrikaew, & Knoczak, 2021), presenting many nutritional health benefits, including anti-oxidation, liver protection, and atherosclerosis prevention (Sankhari, Thounaojam, Jadeja, Devkar, & Ramachandran, 2012). More than 30 anthocyanin molecules have been identified in RC, mainly including the derivatives of cyanidin-3-diglucoside-5-glucoside (C3dG5G) in non-acylated, mono-acylated, and di-acylated forms (Ghareaghajlou, Hallaj-Nezhadi, & Ghasempour, 2021). However, RC anthocyanins are readily degraded during the processes that occur due to increasing pH, storage time, and temperature (Walkowiak-Tomczak & Czapski, 2007). Therefore, it is necessary to investigate the effect of drying on the anthocyanins in RC.

**Fig. 1:**
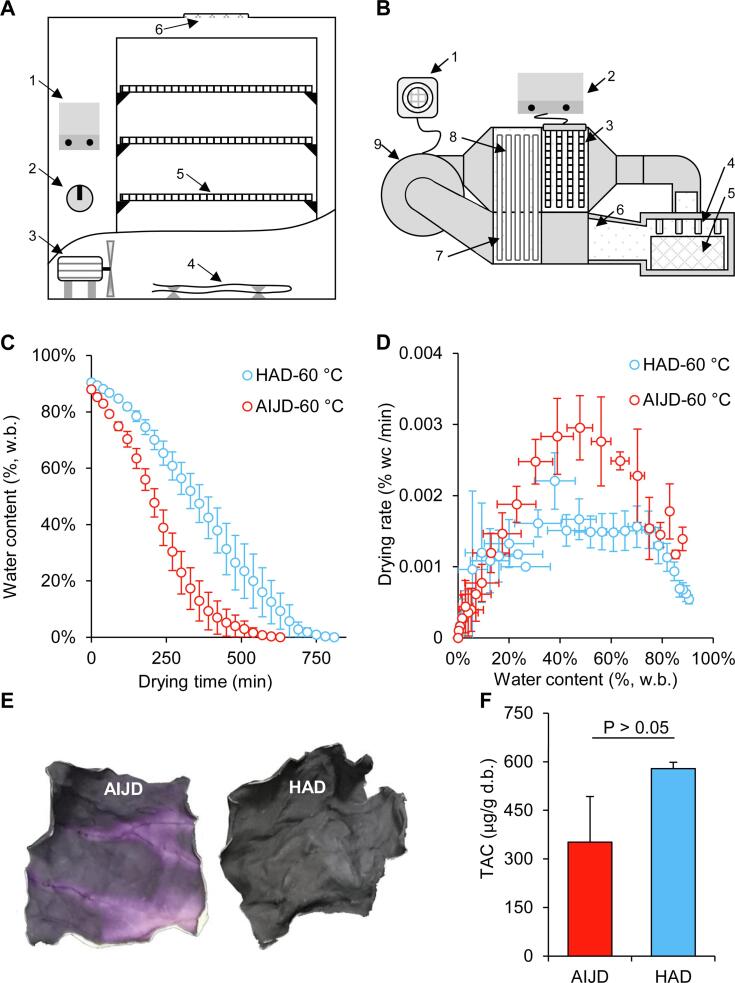
The effect of (A) HAD and (B) AIJD on the (C) drying time, (D) drying rate, (E) appearance, and (F) TAC. (1)-(6) in image A refer to the temperature controller, power switch, electric fan, heater strip, sample network tray, and vent hole, respectively. (1)-(9) in image B denote the air velocity conditioner, temperature controller, electric heating unit, series of circular nozzles, material steel wire mesh box, recovery channel of waste air, the endothermic portion of the heat pipe exchange, the exothermal portion of the heat pipe exchanger, and centrifugal fan, respectively. Both the AIJD and HAD processes were executed at 60 °C. P < 0.05 was considered significantly different. % w.b. refers to the percentage of water per unit of the wet sample. % wc/min signifies the decreased level in the proportion of water per unit wet sample during 1 min of drying.

Among a variety of drying techniques, hot air drying (HAD, Fig. 1A) is the most commonly used for drying fruits and vegetables. However, it is time-consuming and results in serious product quality deterioration (Talens et al., 2017, Zhao et al., 2014). Consequently, air-impingement jet drying (AIJD, Fig. 1B) was developed based on traditional HAD (Luo et al., 2021) in an attempt to improve its disadvantages. Originally, AIJD was used for heat dissipation in electrical components, as well as drying in the paper and textile industries, and it is only recently that it has been applied in food preservation (Li et al., 2015, Xiao et al., 2010). Increasing evidence suggests that AIJD is an efficient drying technique for a variety of fruits and vegetables, such as onions, seedless grapes, and shiitake mushrooms (Bai et al., 2013, Deng et al., 2020, Li et al., 2015, Luo et al., 2021). During AIJD processing, a centrifugal fan transports air through the exothermal portion of a heat pipe exchanger for preheating, where it is further heated by an electric heating unit to the preferred temperature (Li et al., 2015). The residual heat in the waste air is recycled by the endothermic portion of the heat pipe exchanger (Li et al., 2015). An additional advantage of AIJD over HAD is the higher rate of heat and mass transfer (Sarkar, Nitin, Karwe, & Singh, 2004). AIJD reduces the thickness of the boundary layer of heat and mass transfer by jetting air directly onto the surface of the product at a high velocity (10 times that of HAD) (Luo et al., 2021). Therefore, in terms of drying efficiency, AIJD presents a promising alternative for the preservation of RC, although this technique has also been found to induce anthocyanin degradation in purple potatoes and red radishes during the early stages of drying (Qiu et al., 2018, Li et al., 2020).

Kinetic modeling is a useful tool to describe and predict the influence of heat on the degradation of anthocyanins (Zhou, Chen, Bi, Wang, & Wu, 2017). Kinetic analysis has been applied to research the loss of color in RC anthocyanins in a neutral solution (Fenger, Moloney, Robbins, Collins, & Dangles, 2019), as well as the copigmentation of RC polyphenols and anthocyanins in solutions with different pH levels (Parisa, Reza, Elham, & Rashid, 2007). However, the degradation kinetics of the anthocyanins in RC could differ significantly from those in RC juice since solid food samples are capillary porous models while liquid materials are non-porous models (Zhou et al., 2017). Therefore, it is necessary to investigate the degradation kinetics of anthocyanins to minimize their loss in RC during AIJD.

This study aims to reveal the kinetic characteristics of anthocyanin degradation in RC during AIJD. Therefore, the drying efficiency, appearance, and total anthocyanin content (TAC) in RC exposed to AIJD and HAD are compared. The effect of AIJD at different temperatures on the drying efficiency, appearance, TAC, and individual anthocyanin content is investigated. Furthermore, the kinetic and thermodynamic characteristics of RC anthocyanin degradation during AIJD are subsequently revealed.

## Materials and methods

### Materials

The RC (*Brassica oleracea* L. var. *capitata*) was purchased from an agricultural market near Yangtze Normal University. The initial moisture content of the RC sample was 89 ± 1 % (wet basis). Cyanidin chloride (pure > 98 %) and cyanidin-3,5-diglucoside chloride (pure > 95 %) were obtained from Shanghai Yuanye Biotechnology Co., ltd. (Shanghai, China). The methanol (chromatographic purity) was purchased from Adamas Reagent ltd. (Shanghai, China).

### Drying treatment

The RC was cut into slices of approximately 5 cm × 5 cm. The pretreated RC samples (92–94 g) were placed in a single layer on a stainless mesh tray and exposed to HAD at 60 °C and an air speed of 0.6 m/s (ZFD-5040, Shanghai Zhicheng Analytical Instrument Manufacturing Co., ltd., Shanghai, China). The AIJD device used in this study was developed in our laboratory, as described by Li et al. (2015, Fig. 1B). Based on a previous drying study involving Chinese cabbage (Managa, Sultanbawa, & Sivakumar, 2020), the drying temperatures for the AIJD in this current work were set at 50 °C, 60 °C, and 70 °C, at an air velocity of 8 m/s. The samples were weighed to determine the moisture content differences when the weight decrease between two consecutive measurements was below 0.05 g. The drying kinetics results were used to select sample AIJD times for anthocyanin analysis as follows: 0 min, 150 min, 300 min, 480 min, and 720 min at 50 °C, 0 min, 120 min, 240 min, 420 min, and 630 min at 60 °C, and 0 min, 60 min, 150 min, 240 min, and 450 min at 70 °C. Fresh RC was weighed and dried after each sampling. The dried RC samples were subsequently packed into polyethylene bags and stored at −20 °C.

### Measurement of the individual anthocyanins in the RC

A 2 g RC sample (fresh and dehydrated RCs) was cut into small pieces (1 × 1 × 1 mm) and pulped with 10 mL of a 40 % ethanol–water solution for 3 min using a high-speed homogenizer (FSH-2A, Changzhou Jintan Liangyou Instrument Co. ltd., Changzhou, China), after which the homogenate was centrifuged at 2500 × *g* for 10 min. The liquid supernatant (1 mL) was filtrated through an ultrafiltration membrane (0.22 μm) and subjected to analysis via ultra-high performance liquid chromatography coupled with triple quadrupole mass spectrometry (UHPLC-QqQ-MS/MS).

The anthocyanin extract (5 μL) was injected into a UHPLC system (1290II, Agilent, Waldbronn, Germany) equipped with a C18 column (ZORBAX Eclipse plus, 100 × 2.1 mm i.d., 1.8 μm; Agilent, Waldbronn, Germany). The chromatography elution was performed at 40 °C and a flow rate of 0.2 mL/min. Flow phases A and B consisted of a 1 % formic acid–water solution and 1 % formic acid–methanol solution, respectively. The gradients were as follows: 30 %–80 % B from 0 min to 10.5 min; 80 %–95 % B from 10.5 min to 10.6 min; 95 %-95 % B from 10.6 min to 11.2 min; and 95 %-30 % B from 11.2 min to 11.5 min. After each sample determination, the column was balanced for 3.5 min.

Triple quadrupole mass spectrometry (QqQ-MS/MS) (6460C, Agilent, Waldbronn, Germany) was used for data collection. The anthocyanins in the RC were comparatively identified according to previously reported data on molecular ions and fragment ions (Table S1, Wiczkowski, Szawara-Nowak, & Topolska, 2013). The drying and collision gases used in this study were low purity and high purity nitrogen, respectively. The capillary, fragmentor, and collision energy voltages were 3000 V, 130 V, and 30 V, respectively. The cyanidin (C) was quantified using real cyanidin chloride, while the other cyanidin glucosides were relatively quantified using cyanidin-3,5-diglucoside chloride since standard anthocyanins are difficult to obtain. The anthocyanin content was semi-quantitatively calculated as a microgram of anthocyanins per gram of dried matter (μg/g d.b., dried base). The TAC represented the sum of all the monomer anthocyanins.

### Determination of the kinetic and thermodynamic parameters

The kinetic modeling of alterations in food can result in a better molecular-level understanding of what is observed in foods (van Boekel, 2008). In this work, the degradation of the key anthocyanins in the RC was fitted according to 0-, 0.5-, 1-, 1.5-, and 2-order kinetics (Eq. (1)–(5), van Boekel, 2008). Generally, the best model showed a coefficient of determination (R^2^) close to “1″ (Singhal, Rasane, Kaur, Singh, & Gupta, 2020).

(1) $${\text{C}}_{\text{t}}-{\text{C}}_{\text{0}}\text{+kt=0}$$

(2) $$\sqrt{{\text{C}}_{\text{0}}}-\text{2}\sqrt{{\text{C}}_{\text{t}}}\text{+kt=0}$$

(3) $$\text{ln}\left({\text{C}}_{\text{t}}/{\text{C}}_{\text{0}}\right)\text{+kt=0}$$

(4) $$\text{2}\left({{\text{C}}_{\text{0}}}^{-\text{0.5}}-{{\text{C}}_{\text{t}}}^{-\text{0.5}}\right)\text{+kt=0}$$

(5) $$\text{1}/{\text{C}}_{\text{0}}-\text{1}/{\text{C}}_{\text{t}}\text{+kt=0}$$

where C_t_ and C_0_ are the anthocyanin contents (μg/g d.b.) at 0 min and any time of drying, respectively, while t and k are the drying time (min) and rate constant (min^−1^), respectively, of degradation.

The half-life values (t_1/2_), which refer to the time required for a 50 % degradation of the original content (Moon, Pan, & Yoon, 2015), were calculated using Equations (6), (7), below, based on 1.5-order and 2-order kinetics, respectively. The Q_10_-value represents the dependence of a reaction on temperature as the factor by which the reaction rate is altered when the temperature is elevated by 10 °C (van Boekel, 2008). Equation (8) was used to calculate the Q_10_-value for the anthocyanin degradation in the RC during AIJD.

(6) $$t_{1/2}=2\left(\sqrt{2}-1\right)/\left(k\times \sqrt{C_{0}}\right)$$

(7) $$t_{1/2}=1/\left(k\times C_{0}\right)$$

(8) $$Q_{10}=\frac{k_{T+5}}{k_{T-5}}\approx \frac{k_{T+10}}{k_{T}}$$

The temperature dependence of reaction rates can also be explained by the Arrhenius equation (Singhal et al., 2020). Here, a linearized form of the Arrhenius equation (Eq. (9)) was used to analyze the activation energy (Ea, kJ/mol).

(9) $$\text{ln}\text{k}\text{=}\text{ln}\text{A}-\frac{{\text{E}}_{\text{a}}}{\text{RT}}$$

where A is a so-called ‘pre-exponential’ factor and R is the gas constant.

The enthalpy changes (ΔE, kJ/mol), Gibbs free energy (ΔG, kJ/mol), and entropy (ΔS, kJ/mol·K) were calculated using Equations 10, 11, and 12, respectively (Zhou et al., 2017).

ΔH=Ea-RT

$$\Delta \text{G}=-\text{RT}\ln\left(\text{kh}/{\text{k}}_{\text{B}}\text{T}\right)$$

$$\Delta \text{S}=\left(\Delta \text{H}-\Delta \text{G}\right)/\text{T}$$

where h and k_B_ are the Planck constant (6.6262 × 10^-34^ J/s) and Boltzmann constant (1.3806 × 10^-23^ J/K), respectively.

### Statistical analysis

Multivariate analysis is used as a statistical technique to examine the effect of drying on food polyphenols (Li et al., 2019, Li et al., 2020). Therefore, this study used sparse partial least-squares discriminant analysis (sPLS-DA) to reveal the key RC anthocyanins affected by AIJD. For this, the dimensionality reduction relationship among the samples was presented via a score plot of the first two principal components. The “*plotLoadings*” function provided a visualization of the highest contribution value of the variables that could be used to distinguish the differences among the samples. All the multivariate analytical procedures were performed using the R package known as “mixOmics” (https://mixomics.org/). The statistical differences between two groups were analyzed via a Student’s *t*-test, while those among more than two groups were estimated using Tukey’s multiple comparison post-hoc test. The variance analysis was executed using the “agricolae” package (https://CRAN.R-project.org/package=agricolae). The R program used was the 4.1.2 version (https://www.r-project.org/). Furthermore, *P* < 0.05 was considered statistically significant. All the experiments were performed in triplicate.

## Results and discussion

### AIJD is superior to HAD for drying RC

To highlight the technical advantages of AIJD over HAD for drying RC, the impact of both processes on the drying kinetics and drying quality of the RC samples were compared. As shown in Fig. 1A, it took 810 min to sufficiently reduce the moisture content in the RC using HAD at 60 °C, while the AIJD process lasted 630 min. This result highlights the higher drying efficiency of AIJD on RC, which can be attributed to a higher heat and mass transfer rate (Sarkar et al., 2004). Similar results have been widely reported in previous studies (Tan et al., 2021, Li et al., 2015, Li et al., 2020, Li et al., 2020). Although some studies have found that the drying rate curves of AIJD agricultural products showed mainly falling-rate periods (Liu et al., 2019, Xiao et al., 2010), the drying rate curve of the AIJD RC in this study showed unusual elevating-rate periods (Fig. 1B). The changes in the drying rates from elevating-rate periods to falling-rate periods indicate an alteration in the mass transfer mechanism from surface diffusion to internal diffusion as the main moisture transfer pathway (Li. Li et al., 2020). These changes in the RC occur later during the HAD process than AIJD. This phenomenon may also indicate that the drying rate in AIJD was faster than in HAD.

Our previous research showed that AIJD provided a significant advantage in protecting the color of kiwis and onions (Huang et al., 2017, Li et al., 2015). In the current study, the AIJD-treated RC retained its distinct reddish-purple color (Fig. 1C). However, the RC exposed to HAD presented a dark purple color (Fig. 1C). The color variation in dark fruits and vegetables during drying can be attributed to the degradation of anthocyanins (Sun, Zhang, Xu, & Zheng, 2020). The TACs in AIJD- and HAD-treated RC, respectively, were 351.80 ± 141.05 and 579.08 ± 19.24 μg/g d.b. despite the absence of significant difference in these TACs (Fig. 1D). These results suggest that AIJD is a better technique than HAD for drying RC due to its higher drying efficiency and color retention capability. Further improvement of the anthocyanin content in RC exposed to AIJD would help promote its commercial application. Therefore, the effect of AIJD on the anthocyanins in RC was explored.

### AIJD changes the drying time, TAC, and color of RC

**Fig. 2:**
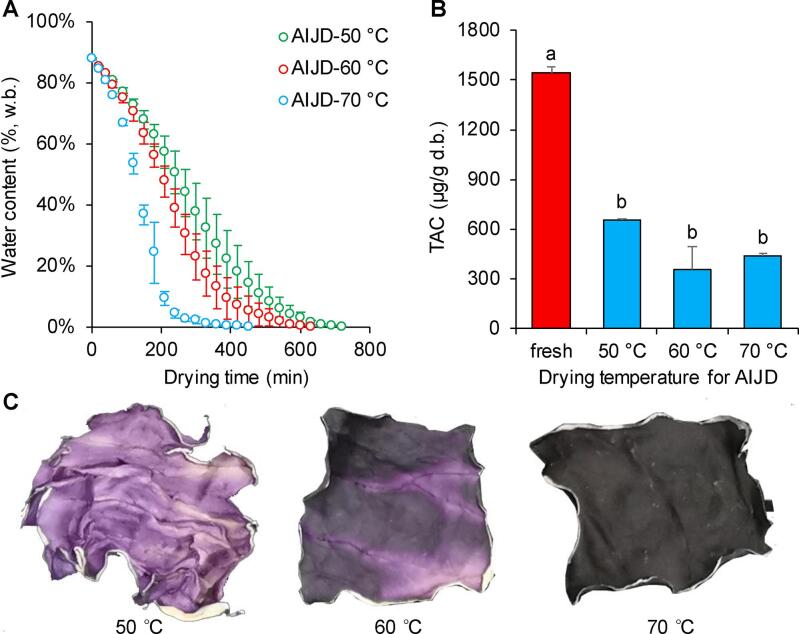
The effect of AIJD at 50 °C, 60 °C, and 70 °C on (A) drying time, (B) appearance, and (C) TAC. Different letters on the bar graph indicate significant differences (P < 0.05). % w.b. refers to the percentage of water per unit of the wet sample. μg/g d.b. shows the micrograms of total anthocyanins per gram of dried sample.

Similar to previous studies (Deng et al., 2020, Liu et al., 2019), the higher drying temperature in this study resulted in a shorter RC drying time. As shown in Fig. 2A, the AIJD times required to achieve the final moisture content level of the RC were 720 min, 630 min, and 450 min at temperatures of 50 °C, 60 °C, and 70 °C, respectively. These drying times were shorter than those required (1260–3060 min) for the AIJD of Monukka seedless grapes at 50–65 °C (Xiao et al., 2010) but longer than those required (90–150 min) to dry orange peel at 50–70 °C (Deng et al., 2020). Although a high drying temperature reduced the drying time, it degraded the active antioxidant compounds, such as vitamin C (Liu et al., 2019), anthocyanins (Qiu et al., 2018), and polyphenols (Deng et al., 2020). This study also revealed that AIJD at 50–70 °C remarkably decreased the TAC by 57 %-77 % (Fig. 2B), from 1546.18 ± 33.30 μg/g d.b. of fresh RC, albeit lower than the AIJD-induced degradation of anthocyanins in RC (81 %-89 %) reported by Li, Pang et al. (2020). Although no statistical differences were evident between the changes in TAC of the three AIJD RC samples in this study, it is worth noting that the TAC in the RC exposed to AIJD at 60 °C (351.81 ± 141.05 μg/g d.b.) was slightly lower than that dried at either 50 °C (651.93 ± 12.62 μg/g d.b.) or 70 °C (437.67 ± 14.20 μg/g d.b., Fig. 2B). Similarly, the TAC in purple potato samples exposed to AIJD at 50–80 °C did not reduce with an increase in temperature (Qiu et al., 2018). This may be because the degradation of anthocyanins is simultaneously influenced by heating, pH, light, oxygen, and exposure time in these conditions (Park et al., 2016, Patras et al., 2010). Changes in anthocyanin content and molecular structure commonly induce variations in color (Danişman, Arslan, & Toklucu, 2015). However, the color in this study was obviously different in the RC exposed to AIJD at 50–70 °C (Fig. 2C), while the TAC of the samples was fairly similar (Fig. 2B). Specifically, the degree of browning in the RC was enhanced by an increase in the AIJD temperature (Fig. 2C). These results indicate that it may not be reasonable to use TAC alone as a reflection of the effect of drying on the anthocyanins of fruits and vegetables.

### Anthocyanin profile of RC and the main anthocyanins affected by AIJD

**Fig. 3:**
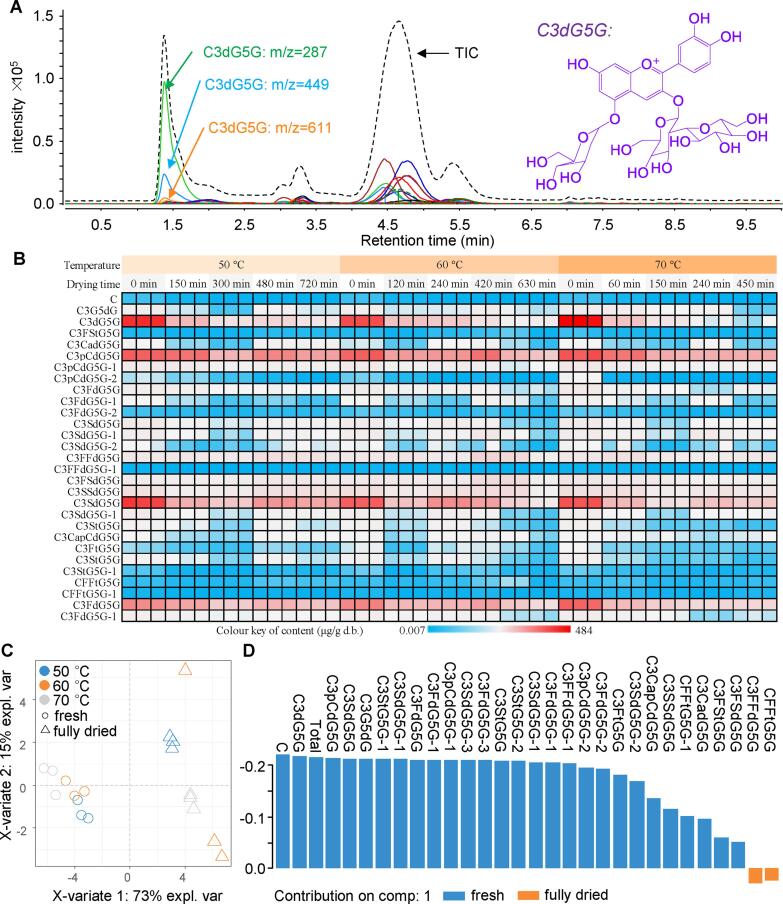
AIJD degraded the individual anthocyanins in the RC. (A) A chromatogram of the total ion signal (TIC) and multiple response monitoring (MRM) of individual anthocyanins in the RC. (B) A heat map of the individual anthocyanin content in the RC. (C) A score plot of the sPLS-DA of the individual anthocyanins. (D) The loading values of the individual anthocyanins on the first principal component of sPLS-DA. Note: C3dG5G refers to cyanidin-3-diglucoside-5-glucoside. The 1st-5th in the sampling order indicates sampling with increasing drying time; 50 °C for 0 min, 150 min, 300 min, 480 min, and 720 min; 60 °C for 0 min, 120 min, 240 min, 420 min, and 630 min; 70 °C for 0 min, 60 min, 150 min, 240 min, and 450 min.

UHPLC-QqQ-MS/MS was used to qualitatively and quantitatively analyze the effects of AIJD on the individual anthocyanins in RC (Fig. 3A). As shown in Table S1, one anthocyanidin and 28 anthocyanins were identified in the RC samples. Interestingly, the same mass spectrometry information revealed multiple peaks with different retention times (RT), such as cyanidin-3-(p-coumaroyl)-diglucoside-5-glucoside (C3pCdG5G), cyanidin-3-(feruloyl)-diglucoside-5-glucoside (C3FdG5G), and cyanidin-3-(sinapoyl)-diglucoside-5-glucoside (C3SdG5G) (Table S1). This may be because the acylated glycosidic structure induced cis–trans isomers, which can have different RTs in the same chromatographic conditions (Charron, Clevidence, Britz, & Novotny, 2007). Similar results were obtained by previous studies on anthocyanins in purple cabbage (Charron et al., 2007, Wiczkowski et al., 2013). The results of the current study concur with those reported by Müller-Maatsch, Gurtner, Carle, and Björn Steingass (2019), in which anthocyanins in RC were found mainly to derive from C3dG5G. In our study, C3dG5G was also the main anthocyanin in the RC, accounting for 25.25 % ± 1.99 % of TAC, which corresponded with the result of 25 % previously reported by Wiczkowski et al. (2013). Moreover, C3pCdG5G, C3SdG5G, and C3FdG5G were also found in high proportions in the RC in our study (Fig. 3B).

To expose the main effect of AIJD on individual anthocyanins in the RC, a data matrix comprising all anthocyanin contents was submitted to sPLS-DA analysis. As illustrated in Fig. 3C, the first and second principal components could explain 73 % and 15 % of variates, respectively. Fresh and fully dried samples can be seen on either side of the first principal component, suggesting that the first principal component effectively differentiated between the fresh and fully dried RC (Fig. 3C). Consequently, the loading values of all anthocyanins on the first principal component were analyzed (Fig. 3D). The results indicated that C3dG5G and C presented higher loading values than the other anthocyanins, suggesting that these RC compounds were affected the most by AIJD. Accordingly, the C3dG5G and C content in the RC decreased by 71.74 % ± 6.70 % and 60.52 % ± 2.46 %, respectively, during the 0–60 min drying process. Therefore, the degradation kinetics and thermodynamic parameters of C3dG5G and C were selected for further exploration.

### Degradation kinetics and thermodynamic parameters of C3dG5G and C

According to Zhou et al. (2017), a kinetic equation used to describe and predict the degradation behavior of anthocyanins should have a high coefficient of determination (R^2^). Since 1.5-order and 2-order kinetic equations have higher R^2^ than other kinetic equations, they were selected to describe and predict the degradation of C3dG5G and C, respectively (Table 1). Although this result differed from that 1-order kinetic equation could fit the anthocyanin degradation during purple potato slice AIJD (Qiu et al., 2018), 2-order kinetic model was also found to describe the degradation of anthocyanin during red radish AIJD, and mulberry HAD and vacuum drying (Li et al., 2020, Zhou et al., 2017). Aglycon-sugar bond hydrolysis is generally the first step in anthocyanin degradation, followed by anthocyanidin deterioration (Patras et al., 2010). Although anthocyanidin cleavage is also an important reaction leading to anthocyanidin degradation, it is unlikely to occur in AIJD conditions of 50–70 °C (Patras et al., 2010) and may not be high enough. Previous studies have also confirmed that the degradation patterns of anthocyanins with different molecular structures are distinctly different (Li et al., 2021). Accordingly, the differences between optimal kinetic models may be attributed to the varied response of anthocyanins with different molecular structures to the heat and mass transfer conditions of different drying technologies.

**Table 1:** R^2^ for the kinetic equation simulation of AIJD-induced degradation of anthocyanins in RC.

Anthocyanins	Temperature	0-order	0.5-order	1-order	1.5-order	2-order
C3dG5G	50 °C	0.5861	0.6597	0.7247	0.7551	0.7488
	60 °C	0.6699	0.8219	0.9426	0.9698	0.9428
	70 °C	0.4664	0.6335	0.8420	0.9284	0.9329
C	50 °C	0.6605	0.7603	0.8569	0.9243	0.9550
	60 °C	0.6545	0.7634	0.8640	0.9230	0.9375
	70 °C	0.5738	0.6725	0.7745	0.8617	0.9238

The *k* and *t_1/2_* values were calculated (Table 2), with constant *k* a function of Ea and temperature, expressing the reaction rate (Sun et al., 2020). The results showed that C had a higher *k* and a lower *t_1/2_* than C3dG5G, indicating that C degraded more rapidly than C3dG5G during the AIJD of the RC (Table 2). Although glycosylation, acylation, and methoxylation enhanced the stability of anthocyanins in a solution against pH changes and metal ions (Li et al., 2021, Marszałek et al., 2015), the glycosidic bonds became unstable and broke away from the aglycone during thermal treatment (Patras et al., 2010). Furthermore, the deglycosylated product of cyanidin glycoside is C (Sadilova, Stintzing, & Carle, 2006) and, consequently, the C content in the RC may not decrease as rapidly as C3dG5G during AIJD since it is simultaneously and continuously produced. Furthermore, the *t_1/2_* values of C3dG5G (21.21–141.43 min) and C (30.59–74.53 min) were lower than those of anthocyanins previously reported in a study involving red radishes during AIJD at 50–70 °C (84.80–200.80 min) (Li, Pang et al., 2020). Additionally, the *k* and *t_1/2_* values increased and declined, respectively, in conjunction with a higher drying temperature (Table 2). Similar results were evident in almost all studies on heat-induced anthocyanin degradation (Das et al., 2020, Sun et al., 2020). Accordingly, it can be concluded that the molecular structure and temperature of drying co-regulate the degradation rate of anthocyanins in RC during AIJD.

**Table 2:** The kinetic and thermodynamic parameters of anthocyanin degradation in the RC exposed to AIJD.

Anthocyanins	Temperature	k (min^−1^)	t_1/2_ (min)	Ea (kJ/mol)	ΔH (kJ/mol)	ΔG (kJ/mol)	ΔS (kJ/mol·K)	Q_10_-value
C3dG5G	50 °C	0.0003	8.34	87.19	84.47	101.15	−0.052	50–60 °C: 2.00
	60 °C	0.0006	4.17		84.39	102.45	−0.054	60–70 °C: 3.33
	70 °C	0.0020	1.25		84.30	102.17	−0.052	
C	50 °C	0.0094	74.53	41.21	38.49	91.90	−0.17	50–60 °C: 1.88
	60 °C	0.0177	39.58		38.40	93.07	−0.16	60–70 °C: 1.29
	70 °C	0.0229	30.59		38.32	95.22	−0.17	

k: rate constant; t_1/2_: half-time; Ea: activation energy; ΔH: enthalpy change; ΔG: Gibbs free energy; ΔS: entropy change.

As presented in Table 2, the Ea values of C3dG5G and C were calculated as 87.19 and 41.21 kJ/mol, respectively, indicating that C3dG5G required more energy than C to reach an active state of degradation and was, thus, more likely to degrade than C during the AIJD of RC. Moreover, the Ea values in this study were higher than those reported for the anthocyanins in red radish during AIJD (5.73–39.92 kJ/mol) (Li, Pang et al., 2020), suggesting that the anthocyanins of RC are more sensitive to high temperatures than those of red radishes. However, since three temperatures might not precisely reveal the Ea via the slope method based on the Arrhenius equation (Li, Pang et al., 2020), the Q_10_-value was further calculated, which strongly depended on the temperature (van Boekel, 2008). A Q_10_-value greater than 1 indicated that a reaction was enhanced by increased temperature (Li, Pang et al., 2020). Accordingly, the degradation of C3dG5G and C during the AIJD of RC at 50–70 °C in this study was likely regulated by an elevated temperature since the Q_10_-values of C3dG5G, and C ranged from 2.00 to 3.33 and from 1.29 to 1.88, respectively.

Since ΔH represents the energy difference between a reagent and an activated complex, small ΔH values contribute to the generation of an activated complex due to the low potential energy barrier (Zhou et al., 2017). In this study, all ΔH values exceeded 0 (Table 2), indicating that the degradation of C3dG5G and C during the AIJD of RC were endothermic reactions (Silva, Crispim, & Vieira, 2016). These findings were consistent with earlier reports of anthocyanin degradation during ohmic heating, thermostatic water bathing, HAD, and vacuum drying (Mercali et al., 2015, Silva et al., 2016, Zhou et al., 2017), indicating that the energy barrier that must be overcome to achieve the transition state is similar for all these heating technologies (Mercali et al., 2015).

ΔG represents the difference between an activated state and reactants, with a positive value indicating a nonspontaneous reaction (Silva et al., 2016). In this study, close values for all drying temperatures were evaluated (91.90–102.45 kJ/mol), showing positive ΔG values, suggesting that the AIJD-induced degradation of the anthocyanins in the RC was nonspontaneous. The total energy increase in the system at the approach of the reagents and the formation of the activated complex were similar at all drying temperatures. These ΔG values were close to those previously obtained in a study involving AIJD purple potatoes (103.81–112.33 kJ/mol) (Qiu et al., 2018) and red radishes (89.54–94.90 kJ/mol) (Li, Pang et al., 2020).

The ΔS values indicate molecule disorder in a system (Zhou et al., 2017). The ΔS values for the C3dG5G (−0.054 – −0.052 kJ/mol) and C (−0.17 – −0.16 kJ/mol) in the AIJD RC in this study were negative (Table 2), suggesting that the degradation products might have less structural freedom in the intermediate state (complex) than the reactant, resulting in the presence of an entropy barrier in the system (Singhal et al., 2020). These ΔS values were close to those reported for anthocyanin degradation during the AIJD of red radishes (Li, Pang et al., 2020) but were significantly higher than those reported by Qiu et al. (2018) for purple potatoes exposed to AIJD.

### Prediction of the anthocyanin content using kinetic equations

**Fig. 4:**
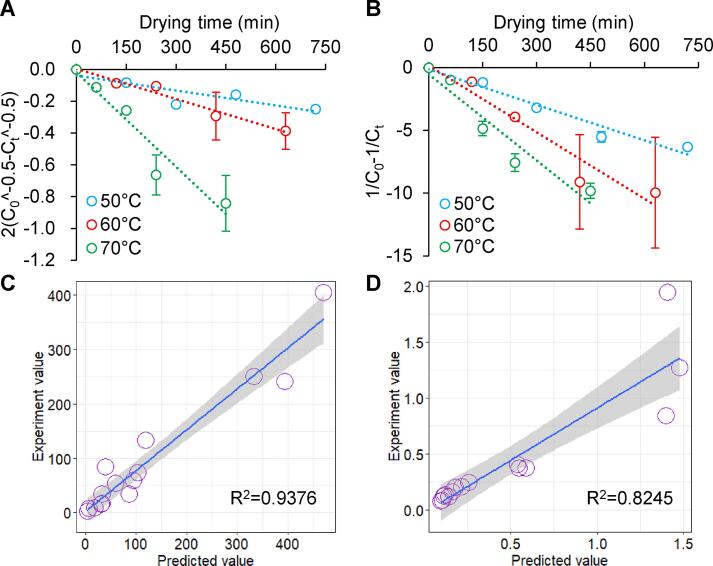
The 1.5-order (A, C) and 2-order (B, C) kinetic equations describe (A, B) and predict (C, D) the degradation of C3dG5G and cyanidin in RC during AIJD. The shaded areas in C and D represent 95 % confidence intervals.

In numerous previous studies, kinetic equations have been used to reveal the kinetic and thermodynamic parameters for the degradation of anthocyanins (Qiu et al., 2018, Mercali et al., 2015, Li et al., 2020, Zhou et al., 2017). As shown in Fig. 4A and B, the anthocyanin content in the RC changed as the period of exposure to AIJD increased but appeared on both sides of the kinetic equation line. Therefore, the optimum kinetic equation can be used to predict the anthocyanin content in the RC during AIJD. Based on the coefficient of the linear regression equation (y = ax + b), the functions of the coefficient and temperature were constructed (Table S2) and subsequently applied to construct prediction equations for C3dg5G (Eq. (13)) and C (Eq. (14)) based on the 1.5-order and 2-order equations, respectively. As shown in Fig. 4C and D, the predicted values banded around the straight line, while the R^2^ exceeded 0.8, indicating that equations (13), (14) could be used to predict the content of C3dg5G and C, respectively, in the RC during AIJD at 50–70 °C. Consequently, these results can be used to improve the AIJD process for RC.

(13) $$2\left(C_{0}^{-0.5}-C_{t}^{-0.5}\right)=\left(-6\times 10^{-6}\times T^{2}+0.0006T-0.0153\right)t+\left[0.0628\ln\left(T\right)-0.2741\right]$$

(14) $$\frac{1}{C_{0}}-\frac{1}{C_{t}}=\left(-0.0007T+0.0238\right)t+\left(-0.0049T^{2}+0.5696T-16.34\right)$$

where T and t represent the drying temperature and time, respectively, and C_0_ and C_t_ denote the initial content and content at any time, respectively, of the anthocyanins.

## Conclusion

The results of this study suggest that AIJD may be a better approach for drying RC than HAD, presenting a higher drying efficiency and retaining superior color. AIJD distinctly changes the RC anthocyanidin profile, which contains 28 cyanins and cyanidins. The 1.5-order and 2-order kinetic equations can be used to describe and successfully predict the degradation behavior of the main compounds, C3dG5G and C, during the exposure of RC to AIJD. Furthermore, the AIJD of RC at high temperatures is not recommended since it enhances C3dG5G and C degradation. These results promote the use of AIJD as a promising technology for drying and preserving RC.

## CRediT authorship contribution statement

**Wenfeng Li:** Conceptualization, Methodology. **Guangfeng Gou:** Investigation. **Yanling He:** Investigation. **Si Tan:** Investigation.

## Declaration of Competing Interest

The authors declare that they have no known competing financial interests or personal relationships that could have appeared to influence the work reported in this paper.
