# Supplementary material for: Innovative air-impingement jet drying of red cabbage: Kinetic description and prediction of the degradation of cyanidin-3-diglucoside-5-glucoside and cyanidin
Source: Food Chem X. 2022 Aug 10;15:100422. doi: 10.1016/j.fochx.2022.100422 (PMC9532788; doi:10.1016/j.fochx.2022.100422)
Supplement: Supplementary data 1 [file mmc1.docx]

**Table S1** Anthocyanins identified in red cabbage

| No. | Abbreviation | Compounds | [M]+ | ms/ms (m/z) | RT (min) |
| --- | --- | --- | --- | --- | --- |
| 1 | C3dG5G | Cyanidin-3-diglucoside-5-glucoside | 773 | 611, 449, 287 | 1.382 |
| 2 | C3G5dG | Cyanidin-3-glucoside-5-diglucoside | 611 | 449, 287 | 4.711 |
| 3 | C3SdG5G | Cyanidin-3-(sinapoyl)-diglucoside-5-glucoside | 979 | 817, 449, 287 | 3.313 |
| 4 | C3SdG5G-1 | Cyanidin-3-(sinapoyl)-diglucoside-5-glucoside | 979 | 817, 449, 287 | 4.769 |
| 5 | C3StG5G | Cyanidin-3-(sinapoyl)-triglucoside-5-glucoside | 1141 | 979, 449, 287 | 3.056 |
| 6 | C3CapCdG5G | Cyanidin-3-(caffeoyl)(*p*-coumaroyl)-diglucoside-5-glucoside | 1081 | 919, 449, 287 | 2.519 |
| 7 | C3FtG5G | Cyanidin-3-(feruloyl)-triglucosides-5-glucoside | 1111 | 949, 449, 287 | 2.803 |
| 8 | C3StG5G | Cyanidin-3-(sinapoyl)-triglucosides-5-glucoside | 1141 | 979, 449, 287 | 1.926 |
| 9 | C3StG5G-1 | Cyanidin-3-(sinapoyl)-triglucosides-5-glucoside | 1141 | 979, 449, 287 | 2.964 |
| 10 | CFFtG5G | Cyanidin-3-(feruloyl)(feruloyl)-triglucoside-5-glucoside | 1287 | 1125, 449, 287 | 3.716 |
| 11 | CFFtG5G-1 | Cyanidin-3-(feruloyl)(feruloyl)-triglucoside-5-glucoside | 1287 | 1125, 449, 287 | 5.436 |
| 12 | C3FdG5G | Cyanidin-3-(feruloyl)-diglucoside-5-glucoside | 949 | 787, 449, 287 | 3.263 |
| 13 | C3FdG5G-1 | Cyanidin-3-(feruloyl)-diglucoside-5-glucoside | 949 | 787, 449, 287 | 4.657 |
| 14 | C3FStG5G | Cyanidin-3-(feruloyl)(sinapoyl)-triglucoside-5-glucoside | 1317 | 1155, 449, 287 | 3.746 |
| 15 | C3CadG5G | Cyanidin-3-(caffeoyl)-diglucoside-5-glucoside | 935 | 773, 449, 287 | 3.711 |
| 16 | C3pCdG5G | Cyanidin-3-(*p*-coumaroyl)-diglucoside-5-glucoside | 919 | 757, 449, 287 | 3.050 |
| 17 | C3pCdG5G-1 | Cyanidin-3-(*p*-coumaroyl)-diglucoside-5-glucoside | 919 | 757, 449, 287 | 4.445 |
| 18 | C3pCdG5G-2 | Cyanidin-3-(*p*-coumaroyl)-diglucoside-5-glucoside | 919 | 757, 449, 287 | 7.040 |
| 19 | C3FdG5G | Cyanidin-3-(feruloyl)-glucoside-5-glucoside | 787 | 449, 287 | 3.255 |
| 20 | C3FdG5G-1 | Cyanidin-3-(feruloyl)-glucoside-5-glucoside | 787 | 449, 287 | 4.721 |
| 21 | C3FdG5G-2 | Cyanidin-3-(feruloyl)-glucoside-5-glucoside | 787 | 449, 287 | 5.504 |
| 22 | C3SdG5G | Cyanidin-3-(sinapoyl)-glucoside-5-glucoside | 817 | 449, 287 | 3.305 |
| 23 | C3SdG5G-1 | Cyanidin-3-(sinapoyl)-glucoside-5-glucoside | 817 | 449, 287 | 4.873 |
| 24 | C3SdG5G-2 | Cyanidin-3-(sinapoyl)-glucoside-5-glucoside | 817 | 449, 287 | 5.484 |
| 25 | C3FFdG5G | Cyanidin-3-(feruloyl)(feruloyl)-diglucoside-5-glucoside | 1125 | 963, 449, 287 | 2.884 |
| 26 | C3FFdG5G-1 | Cyanidin-3-(feruloyl)(feruloyl)-diglucoside-5-glucoside | 1125 | 963, 449, 287 | 5.357 |
| 27 | C3FSdG5G | Cyanidin-3-(feruloyl)(sinapoyl)-diglucoside-5-glucoside | 1155 | 993, 449, 287 | 5.458 |
| 28 | C3SSdG5G | Cyanidin-3-(sinapoyl)(sinapoyl)-diglucoside-5-glucoside | 1185 | 1023, 449, 287 | 5.488 |
| 29 | C | Cyanidin | 287 | 287 | 4.692 |

**Table S2** The experimental and predicted values of regression equation coefficient.

| Anthocyanins | Temperature | experimental a | experimental b | predicted a | predicted b |
| --- | --- | --- | --- | --- | --- |
| C3dG5G | 50 °C | -0.0003 | -0.0379 | -0.0003 | -0.0284 |
|  | 60 °C | -0.0006 | 0.0036 | -0.0009 | -0.0170 |
|  | 70 °C | -0.0020 | -0.0185 | -0.0027 | -0.0073 |
| C | 50 °C | -0.0094 | -0.1357 | -0.0112 | -0.1100 |
|  | 60 °C | -0.0177 | 0.1595 | -0.0182 | 0.1960 |
|  | 70 °C | -0.0229 | -0.5272 | -0.0252 | -0.4780 |

C3dG5G: cyanidin-3-diglucoside-5-glucoside; C: cyanidin. Coefficients of C3dG5G from 1.5-order equation; Coefficients of C from 2-order equation.
